# DDX21 is a p38-MAPK-sensitive nucleolar protein necessary for mouse preimplantation embryo development and cell-fate specification

**DOI:** 10.1098/rsob.210092

**Published:** 2021-07-14

**Authors:** Pablo Bora, Lenka Gahurova, Andrea Hauserova, Martina Stiborova, Rebecca Collier, David Potěšil, Zbyněk Zdráhal, Alexander W. Bruce

**Affiliations:** ^1^ Laboratory of Early Mammalian Developmental Biology (LEMDB), Department of Molecular Biology and Genetics, Faculty of Science, University of South Bohemia, Branišovská 31, 37005 České Budějovice, Czech Republic; ^2^ Laboratory of Biochemistry and Molecular Biology of Germ Cells, Institute of Animal Physiology and Genetics, CAS, Rumburská 89, 27721 Liběchov, Czech Republic; ^3^ Central European Institute of Technology, Masaryk University, 62500 Brno, Czech Republic

**Keywords:** DDX21, p38-MAPK, preimplantation embryo development, cell fate specification

## Abstract

Successful navigation of the mouse preimplantation stages of development, during which three distinct blastocyst lineages are derived, represents a prerequisite for continued development. We previously identified a role for p38-mitogen-activated kinases (p38-MAPK) regulating blastocyst inner cell mass (ICM) cell fate, specifically primitive endoderm (PrE) differentiation, that is intimately linked to rRNA precursor processing, polysome formation and protein translation regulation. Here, we develop this work by assaying the role of DEAD-box RNA helicase 21 (DDX21), a known regulator of rRNA processing, in the context of p38-MAPK regulation of preimplantation mouse embryo development. We show nuclear DDX21 protein is robustly expressed from the 16-cell stage, becoming exclusively nucleolar during blastocyst maturation, a localization dependent on active p38-MAPK. siRNA-mediated clonal *Ddx21* knockdown within developing embryos is associated with profound cell-autonomous and non-autonomous proliferation defects and reduced blastocyst volume, by the equivalent peri-implantation blastocyst stage. Moreover, ICM residing *Ddx21* knockdown clones express the EPI marker NANOG but rarely express the PrE differentiation marker GATA4. These data contribute further significance to the emerging importance of lineage-specific translation regulation, as identified for p38-MAPK, during mouse preimplantation development.

## Graphical abstract

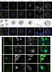

## 1. Introduction

Mammalian preimplantation embryonic development is the period between fertilization and uterine implantation. This period encompasses zygotic genome activation (ZGA), cellular proliferation and lineage specification, culminating in a metabolically active outer trophectoderm (TE) cell layer, fluid-filled blastocoel cavity, epithelial primitive endoderm (PrE) layer and pluripotent epiblast cells (EPI) enveloped between the TE and PrE. Primary transcriptional regulators of cell fate, such as TEAD4 and CDX2 (TE fate), GATA6, SOX17 and GATA4 (PrE fate) and NANOG and SOX2 (EPI fate), have been identified in the mouse model and are enabled by specific signalling networks, namely Hippo-YAP for TE and FGF4-FGFR1/2-MEK/ERK for PrE (as extensively reviewed recently [1–4]). Furthermore, mechanical forces acting on the developing early embryo, on both cellular and embryonal levels, have been found to be significant in the spatial sorting and cell fate specification of individual blastomeres [5–7]. Additionally, a more holistic view of preimplantation embryonic development is emerging by the incorporation of regulatory studies targeting global events, such as translation [8–11]. Indeed, regulation of translation is reported to be vital to modulating pluripotency around the peri-implantation stage [8], enabling embryonic diapause [9] and potentiating global transcriptional regulation [10].

In previous reports, we have described an elementary role for p38-mitogen-activated protein kinase (p38-MAPK) signalling on PrE specification in the mouse blastocyst inner cell mass (ICM) [12], as well as a distinct function mitigating amino acid deprivation-induced oxidative stress during blastocyst maturation [13]. Recently, we extended these investigations and described an early blastocyst functional role in regulating ribosomal RNA (rRNA) processing, translation and transcription required for correct mouse blastocyst expansion and PrE cell fate specification and differentiation. Moreover, that this pathway appears largely independent of the quintessentially described FGF4-based PrE specification mechanisms and is at least partially upstream of the translation regulator mTOR [11]; itself independently implicated in PrE-specific survival in late (E4.5) blastocysts [14]. p38-MAPK activity is also necessary for functional TE derivation prior to blastocyst formation, and its inhibition (p38-MAPKi) from the 16-cell stage (E3.0) specifically impairs fluid-filling of the blastocoel cavity by functionally impacting tight-junction proteins (TJP1), aquaporins (AQP3) and Na^+^/K^+^ pumps (such as ATP1); possibly as a result of p38-MAPKi-induced translational and transcriptional deregulation [11,15,16]. Additionally, p38-MAPKi from the 8-cell stage (E2.5) is reported to cause developmental arrest, at the 8- to 16-cell stage transition, and is associated with defective embryo compaction and impaired filamentous actin formation [17].

In our recent publication describing the role of p38-MAPK in protein translation regulation and PrE differentiation, we reported the results of a phosphoproteomics screen for differentially expressed phosphoproteins in early mouse blastocysts ± p38-MAPKi; aimed at identifying relevant potential p38-MAPK effectors/substrates [11]. This analysis identified the Myb-binding protein 1A (MYBBP1A), a known regulator of rRNA transcription and processing [18] (shown by us to be defective in p38-MAPKi blastocysts [11]). We showed siRNA-induced clonal downregulation of *Mybbp1a* was associated with cell-autonomous proliferation defects and a strong bias against PrE differentiation within mouse blastocyst ICM [11]. The HIV Tat-specific factor 1 homologue (HTATSF1) protein, itself implicated in genetic knockout studies as regulating peri-implantation stage blastocyst EPI pluripotency [8], is a known functional interacting partner of MYBPP1A [8,19]. HTATSF1 also functionally associates with DEAD-box RNA helicase 21 (DDX21) [8,20]. Interestingly, although DDX21 was not specifically identified in our published ± p38-MAPKi early blastocyst screen [11] (falling outside the prescribed filters), we had previously observed it as a differentially phosphorylated protein in our preliminary optimization trials (electronic supplementary material, table S1). Therefore, we decided to revisit the (to date uncharacterized) functional role of DDX21 during mouse preimplantation development within the context of p38-MAPK activity.

DDX21 (initially termed RNA helicase II/Guα) is primarily, but not exclusively, a nucleolar-localized RNA helicase with ATPase activity, shown to be a regulator of rRNA processing; downregulation of which causes decreased production of both 18S and 28S rRNA and accumulation of unprocessed 20S pre-rRNA transcripts [21,22]. It co-fractionates as a protein complex consisting of pre-60S ribosomal subunits and other rRNA processing proteins, such as Pescadillo homologue 1 (PES1), Eukaryotic translation initiation factor 4E-binding protein 2 (EIF4EBP2) and Guanine nucleotide-binding protein-like 3 (GNL3; also known as Nucleostemin/NS) [23]. Recent detailed molecular studies have reported diverse DDX21 roles that range from rRNA metabolism and regulation of RNA polymerase (pol) I and II-mediated transcription to nucleotide stress response and c-Jun activity [20,24–27]. Indeed, DDX21 regulates RNA pol II-mediated transcription of mRNA and non-coding RNA components of ribonucleoprotein complexes [20]. According to another recent report, DDX21 interacts with a specific long non-coding (lnc) RNA, termed the ‘small nucleolar RNA (snoRNA)-ended lncRNA that enhances pre-rRNA transcription’ or SLERT, to reduce its inhibitory affinity for the RNA pol I complex [26]. Regulation of DDX21 activity itself is under the control of post-translational modification, with CREB-binding protein (CBP)-mediated acetylation and Sirtuin7 (SIRT7)-mediated deacetylation, respectively, decreasing or increasing its helicase activity [28]. While some, rRNA metabolism-related mouse genes, for example, *Mybbp1a* [18], *Gnl3* [29] and potentially *Htatsf1* [8], are associated with embryonic lethal genetic knockout phenotypes, no similar *Ddx21-*specific studies are reported. However, the Mouse Genome Informatics (MGI) database does catalogue evidence that *N*-ethyl-*N*-nitrosourea (ENU)-induced mutagenesis of the *Ddx21* gene is embryonically lethal (http://www.informatics.jax.org/marker/MGI:1860494). Ribosomopathies, a collective term for varied human congenital developmental disorders, are mostly associated with heterozygous mutations in factors involved in ribosome biogenesis [30,31]. Interestingly, experimental perturbation of disease-causing candidate genes, such as those identified in Treacher Collins syndrome (TCS), Diamond–Blackfan anaemia (DBA) and Shwachman–Diamond syndrome (SDS), have been reported to result in shifted localization of DDX21 from nucleoli to the general nucleoplasm, with associated changes in DDX21 target chromatin interaction and rRNA processing [24]. Additionally, DDX21 can form a complex with the serine/threonine phosphoprotein phosphatase (PPP) family protein PP1 [32], that along with other family members are well characterized late M-phase/cytokinesis cell cycle regulators [33].

Here, using pharmacological inhibition, siRNA-mediated downregulation, confocal immunofluorescence microscopy and image analysis, we report our findings on the role of DDX21 during mouse preimplantation embryo development. We find basal levels of nuclear DDX21 protein expression until the 16-cell (E3.0) stage, when DDX21 levels begin to increase and become robustly expressed in the early (E3.5) blastocysts before becoming nucleolar by the late blastocyst stage. Moreover, that DDX21 protein expression is sensitive to p38-MAPKi during the period of blastocyst maturation (E3.5–E4.5), as evidenced by varying degrees of decreased nucleolar retention. Specific siRNA-mediated targeting of the *Ddx21* gene leads to efficient global mRNA knockdown and associated clonal cell-autonomous reductions in DDX21 protein expression that cause a 50% reduction in the volume of the late blastocyst. Intriguingly, such clonal *Ddx21* downregulation both cell autonomously (within the siRNA-treated clone) and non-cell autonomously (affecting non-microinjected cells) impairs blastomere proliferation and results in severe defects in the numbers of EPI and PrE lineages by the late blastocyst stage; compared to equivalent stage non-specific control siRNA-treated groups. These results complement our recent report on the general translational regulatory role of p38-MAPK during blastocyst development [11] and identify DDX21 as a p38-MAPK effector with an apparently essential role in murine preimplantation embryonic development.

## 2. Results

### 2.1. DDX21 localization shifts from nuclear to nucleolar post-cavitation during preimplantation development

**Figure 1.**
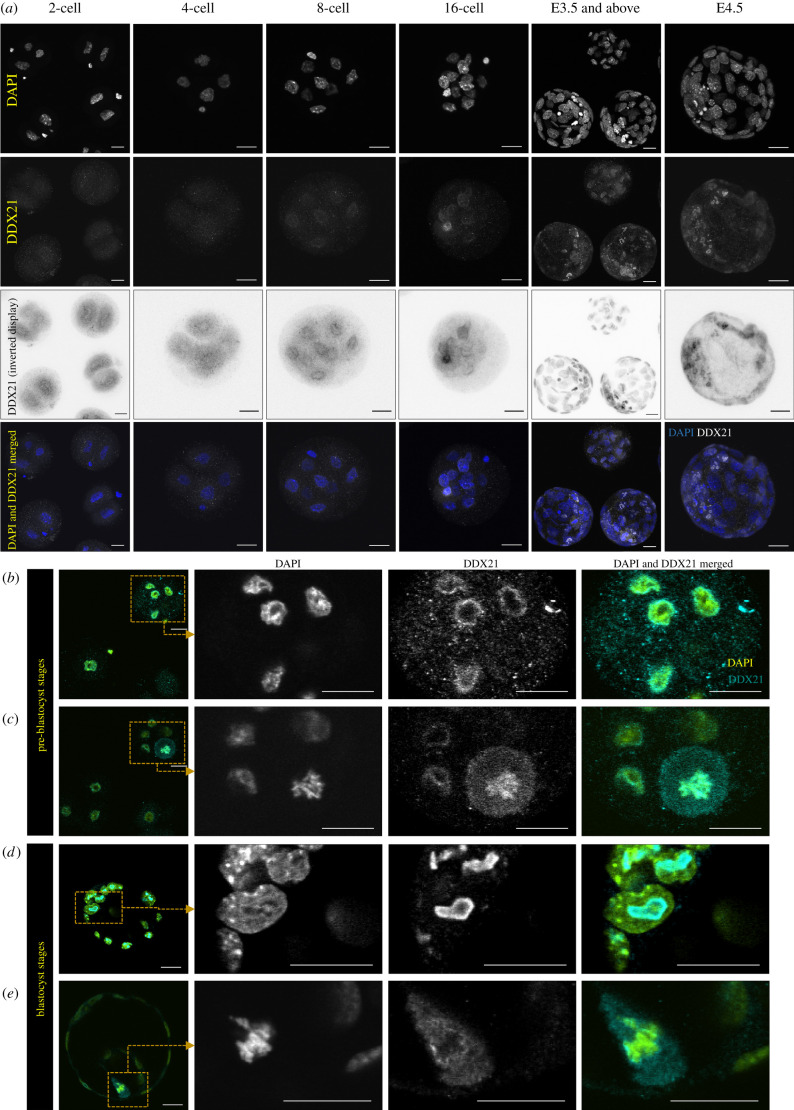
DDX21 protein expression and localization in developing preimplantation mouse embryos. (*a*) From left to right, Z-projection confocal micrographs of 2-, 4-, 8- and 16-cell, E3.5 and E4.5 embryos stained for the nucleus (DAPI), DDX21 (also displayed as an inverted greyscale image) and a merge of the two (DAPI pseudo-coloured blue, DDX21 in greyscale). (*b–e*) Single *z*-section confocal micrograph of an 8-cell stage embryo immunostained for DDX21 with magnified inlay showing (*b*) nucleoplasmic/nuclear membrane localization in interphase cells and (*c*) condensed chromatin localization in a mitotic cell. (*d*) Single *z*-section confocal micrograph of a cavitated blastocyst stage embryo immunostained for DDX21 with magnified image showing nucleolar localization in interphase cells and (*e*) excluded localization from condensed chromatin in a mitotic cell. DAPI and DDX2 signal is pseudo-coloured cyan and yellow in the merged images (*b–e*) and scale bar (*a–e*) = 20 µm.

We previously described an important role of p38-MAPK in coordinating an early blastocyst protein translation response necessary to promote PrE differentiation, identifying MYBPP1A, a known rRNA processing factor, as a p38-MAPK effector [11]. While there is mouse genetics-based evidence implicating some rRNA metabolism-related genes (e.g*. Mybbp1a* [18], *Gnl3* [29] and *Htatsf1* [8]) to critical roles in early development, no similar data exist for the RNA helicase-encoding *Ddx21* gene. Therefore, given the precedents for early developmental functions of similar rRNA-related genes, and the fact we have detected differential phosphorylation of DDX21 protein in mouse blastocysts after p38-MAPKi (electronic supplementary material, table S1), we decided to assay *Ddx21* gene expression throughout the preimplantation developmental stages. Data from recently published transcriptomic studies report *Ddx21* mRNA expression starting to rise from a low steady-state level at the 2-cell (E1.5) stage, peaking at the 8-cell stage and thereafter being maintained at a high level in all preimplantation embryonic cell lineages (electronic supplementary material, figure S1) [34,35]. Immunofluorescent staining of DDX21 protein revealed expression at basal levels in the nuclei of 2-, 4- (E2.0) and 8-cell embryos and robust expression in a subset of 16-cell stage nuclei, that was found in all nuclei by the early through late blastocyst stages, correlating with formation of the blastocyst cavity (figure 1*a*). Interestingly, whereas in pre-cavitated embryos DDX21 protein appeared to be either pan-nucleoplasmic or even adjacently localized to the nuclear membrane (figure 1*b*), post-cavitation, it was found to be exclusively nucleolar (figure 1*d*), suggesting a potential functional significance associated with the onset of blastocyst formation. Similar differential DDX21 localization behaviour regarding mitotic chromatin was also observed. DDX21 readily localized to condensed chromosomes prior to cavitation but appeared to be actively excluded from them post-cavitation (compare figure 1*c*,*e*), further suggesting functional adaptation of the role DDX21 as a response to blastocyst formation.

### 2.2. DDX21 localization is sensitive to p38-MAPK signalling during blastocyst maturation

**Figure 2.**
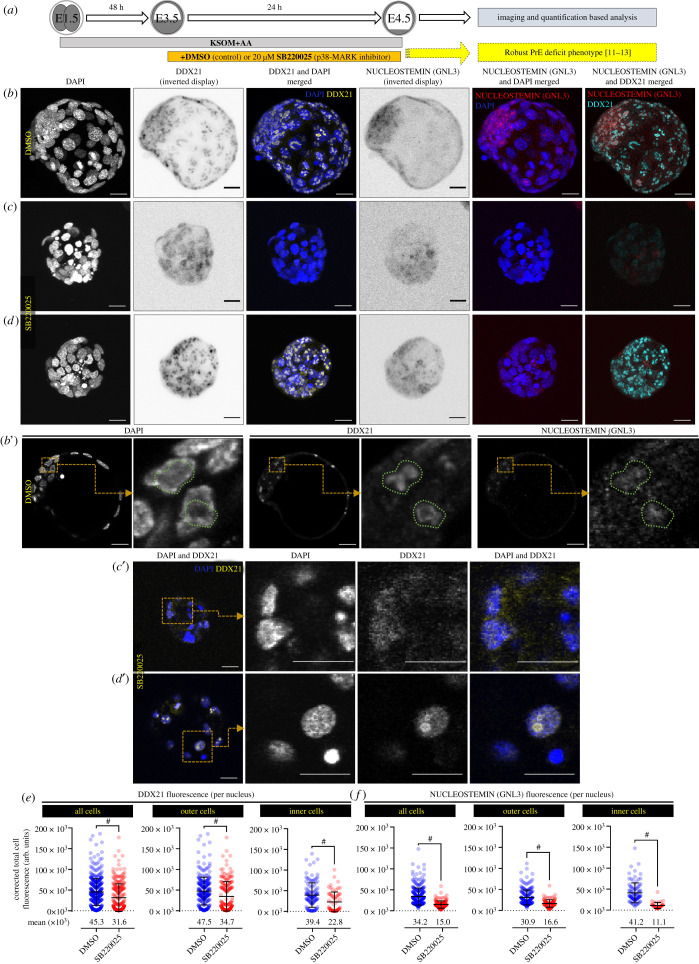
Effect of p38-MAPK inhibition on DDX21 and NUCLEOSTEMIN (GNL3) protein expression and localization during blastocyst maturation. (*a*) Scheme illustrating the experimental protocol of p38-MAPK inhibition (p38-MAPKi) during blastocyst maturation (E3.5–4.5; previously reported by us to be a period during which p38-MAPK signalling is required for PrE specification and blastocyst maturation [11–13]). (*b–d*) Confocal microscopy *z*-projections of (*b*) control (DMSO-treated; *n* = 7) and (*c,d*) p38-MAPKi (SB220025-treated; *n* = 5) embryos immunostained for DDX21 and NUCLEOSTEMIN (GNL3) (both displayed as inverted greyscale images). In DAPI (blue) merged images, DDX21 and NUCLEOSTEMIN signal is pseudo-coloured yellow and red, respectively; in DDX21 and NUCLEOSTEMIN merges, respective cyan and red pseudo-colour pallets are used. (*b′*) Single confocal *z*-sections, with magnified inlays, of DAPI, DDX21 and NUCLEOSTEMIN immunostained nuclei of control (DMSO-treated) blastocysts. Green dotted enclosure demarcates nucleolar localization of DDX21 and the region is superimposed on DAPI and NUCELOSTEMIN images highlighting co-localization. (*c′*,*d′*) Magnified single confocal *z*-sections, with magnified inlays of DDX21 immunostained nuclei (co-stained with DAPI) of p38-MAPKi (SB220025-treated) blastocysts. In merged images, DAPI and DDX21 signals are pseudo-coloured blue and yellow, respectively. Scale bar (*b–d*), 20 µm. (*e*,*f*) Scatter plot quantifications of per cell nuclei corrected total cell fluorescence (CTCF) of (*e*) DDX21 and (*f*) NUCLEOSTEMIN (GNL3) immunostaining in control (DMSO-treated) and p38-MAPKi (SB220025-treated) embryos, as expressed for all cells, outer cells and inner cells. DDX21 : DMSO from *n* = 16 embryos (outer cells = 271, inner cells = 122) and SB220025 from *n* = 15 embryos (outer cells = 190, inner cells = 67) and NUCLEOSTEMIN : DMSO from *n* = 7 embryos (outer cells = 205, inner cells = 97) and SB220025 from *n* = 5 embryos (outer cells = 100, inner cells = 40). Collated individual nuclei CTCF quantifications for (*e*) and (*f*) are provided in electronic supplementary material, data table S1. (Alternative comparative representations of these data are provided in electronic supplementary material, figure S2.)

Collectively, we have previously identified an early blastocyst developmental window of p38-MAPK activity associated with protein translation regulation and required to support PrE differentiation [11], obtained evidence DDX21 is a candidate p38-MAPK substrate (electronic supplementary material, table S1) and revealed blastocyst-specific nucleolar-restricted expression of DDX21 protein. We therefore sought to examine DDX21 protein expression and localization together with that of GNL3 in maturing mouse blastocysts ± p38-MAPKi (E3.5–E4.5; figure 2*a*). GNL3 is a rRNA processing factor functionally related to DDX21 [23], null mutants of which do not develop beyond E4.0 [36,37]. In control E4.5 stage blastocysts, we observed expression of DDX21 and GNL3 in both inner and outer cell nuclei that was co-localized with respect to nucleolar structures (figure 2*b*,*b′* insets). Nevertheless, respective protein expression quantitation revealed DDX21 to be more highly expressed in outer versus inner cell nuclei overall, with the inverse being true for GNL3 (figure 2*e,f*; electronic supplementary material, figure S2). p38-MAPKi resulted in a clear reduction in both DDX21 and GNL3 protein expression that was evident in all blastocyst cells, comprising both inner and outer populations (figure 2*c–f*; electronic supplementary material, figure S2). The percentage decrease upon p38-MAPKi in levels of GNL3 (56% decrease overall) was greater than DDX21 (30% decrease overall); nevertheless, the values were statistically significant in both cases (figure 2*c*–*f*; electronic supplementary material, figure S2). Apart from the changes in general DDX21 protein expression, we also noted p38-MAPKi was associated with a shift in DDX21 protein localization. This was manifest in increased expression in the general nucleoplasm (figure 2*c′*,*d′*) as opposed to exclusively nucleolar expression previously characterized in untreated (figure 1*d*) or control-treated late blastocysts (figure 2*b*,*b′*). We did also observe the degree of p38-MAPKi-dependent change in localization of DDX21 protein away from the nucleolus was heterogeneous (compare figure 2*c*,*c′* with 2*d*,*d′*). This is possibly related to natural heterogeneity in the developmental timings of individual blastocysts at the point of administering p38-MAPKi. Notwithstanding, these data demonstrate the continued general expression of DDX21 and more profoundly GNL3 is sensitive to active p38-MAPK signalling during blastocyst maturation. Additionally, typical blastocyst stage nucleolar localization of DDX21 protein is also regulated by active p38-MAPK. Collectively, these data lend support to the importance of p38-MAPK-related regulation of translation, and particularly rRNA processing, as recently uncovered in early blastocyst maturation and eventual PrE differentiation [11].

### 2.3. Clonal downregulation of *Ddx21* causes reduced embryo cell number and blastocyst expansion

**Figure 3.**
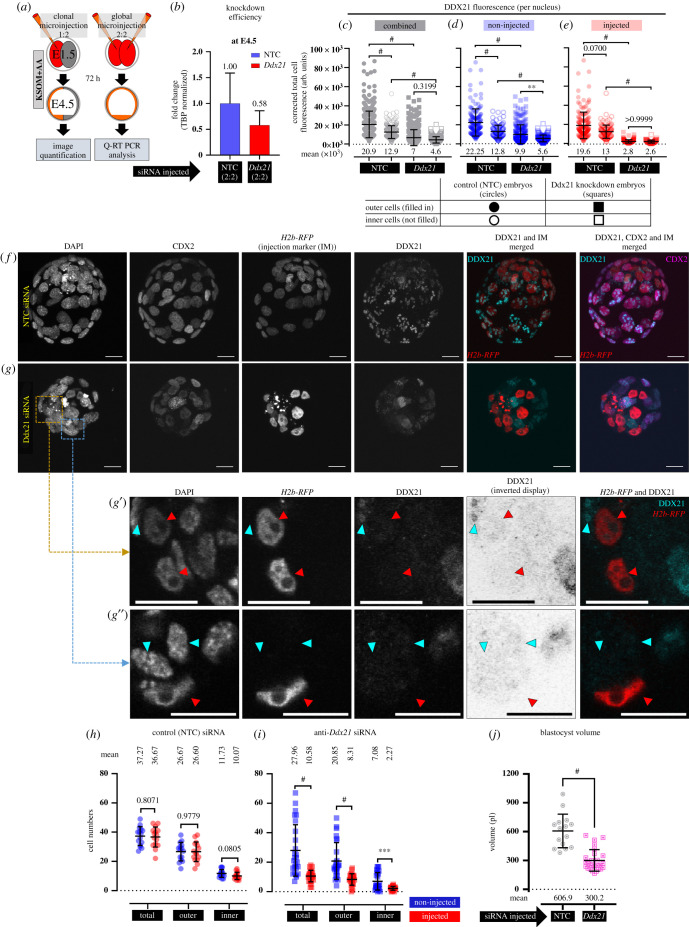
Clonal knockdown of *Ddx21* and effect on late blastocyst morphology and cell numbers. (*a*) Experimental design to determine the efficiency of siRNA-mediated *Ddx21* gene mRNA knockdown in microinjected embryos cultured to the equivalent late blastocyst (E4.5) stage (right panel) and to assay the contribution of marked *Ddx21* knockdown clones to late blastocyst cell lineages (left panel). (*b*) qRT-PCR-derived relative *Ddx21* transcript levels (normalized to *Tbp* mRNA levels) between embryos injected with non-targeting control (NTC) siRNA and siRNA specific for *Ddx21* mRNA at E4.5. (*c*–*e*) Scatter plot quantifications of per cell CTCF values for DDX21 expression in control (NTC siRNA-injected *n* = 15 embryos; non-injected cells outer *n* = 151 and inner *n* = 108, injected cells outer *n* = 153 and inner *n* = 112) and *Ddx21* downregulated (*Ddx21* siRNA-injected *n* = 26; non-injected cells outer *n* = 225 and inner *n* = 125, injected cells outer *n* = 157 and inner *n* = 59) embryos, comparing (*c*) all inner and outer cells, between NTC and *Ddx21* siRNA-injected embryos, (*d*) only non-injected cells between inner and outer cells for both sets of embryos and (*e*) only injected cells between inner and outer cells for both sets of embryos (collated individual nuclei CTCF quantifications for (*c*–*e*) are provided in electronic supplementary material, data table S2; alternative comparative representations of these data are provided in electronic supplementary material, figure S4). (*f*,*g*) Confocal micrograph *z*-projections of representative late (E4.5) stage blastocysts initially microinjected (in one blastomere at the 2-cell stage) with (*f*) NTC siRNA (*n* = 15) and (*g*) *Ddx21* siRNA (*n* = 26), plus recombinant *H2b-RFP* fluorescent reporter mRNA (identifying the clonal progeny of the injected cell). Individual DAPI (greyscale pan-nuclear stain; total number of cells), DDX21 (greyscale) and CDX2 (greyscale; TE cells) channel micrographs, plus merged DDX21 (cyan), CDX2 (magenta) and H2B- RFP (red; microinjected clone) images are shown. (*g*′,*g*″) Magnified single *z*-section confocal micrograph of clonal *Ddx21* downregulated embryo with DDX21 immunostained nuclei representative for both autonomous and non-autonomous effects (and DAPI counterstain). In merged image, H2B-RFP and DDX21 signal is pseudo-coloured red and cyan, respectively. Red arrowheads mark progeny of *Ddx21* siRNA microinjected cells (discernible by H2B-RFP fluorescence—detailing cell-autonomous DDX21 protein knockdown) and cyan arrowhead marks non-microinjected cells detailing in (*g*′) continued but reduced DDX21 expression, or (*g*″) non-autonomous DDX21 protein knockdown). Scale bar (*f–g*″), 20 µm. Scatter plot quantification of cell numbers in (*h*) NTC siRNA and (*i*) *Ddx21* siRNA microinjected embryos, categorized into total, inner and outer cell populations, based on combined CDX2 expression and DAPI fluorescence. Collated individual embryo cell number quantifications are provided in electronic supplementary material, data table S4. (Alternative comparative representations of these data are provided in electronic supplementary material, figure S4.) (*j*) Quantification of blastocyst volume (pl). Collated individual embryo volume quantifications are provided in electronic supplementary material, data table S3.

Given the revealed p38-MAPKi sensitivity of blastocyst DDX21 nucleolar localization, we speculated what the effect of targeted dysregulation of the *Ddx21* gene expression would be on mouse preimplantation embryo development and, given our previously described reports [11–13], on blastocyst ICM cell fate derivation and specifically PrE differentiation. Accordingly, we devised a siRNA microinjection-mediated scheme to downregulate *Ddx21* transcript levels (schematic described in figure 3*a*). To assess *Ddx21*-specific siRNA efficiency, we microinjected both blastomeres of 2-cell stage embryos and quantified normalized *Ddx21* transcript levels at the late blastocyst stage. Compared to non-targeting control (NTC siRNA) microinjected embryos, at the equivalent stage, *Ddx21*-specific siRNA elicited 42% decreased *Ddx21* mRNA expression at E4.5 (figure 3*b*). Although assaying similarly injected embryos at E3.5, we observed an 86% reduction in *Ddx21* mRNA levels (electronic supplementary material, figure S3), demonstrating a possible reduction in the siRNA-mediated knockdown efficiency between E3.5 and E4.5. We next created fluorescently marked embryonic clones by co-microinjecting one cell at the 2-cell stage with siRNA (either NTC or *Ddx21*-specific) and mRNA encoding histone H2B-RFP reporter, thus creating a clone initially comprising 50% of the embryo. Microinjected embryos were then cultured to the late blastocyst stage, fixed, immunofluorescently stained for DDX21 and the outer TE marker protein CDX2 [38] (to distinguish inner and outer cell populations), and the number of cells in the fluorescently marked siRNA microinjected clones, plus non-microinjected sister clones, and their spatial localization within the embryo (outer or inner) recorded.

As shown in the confocal micrographs (figure 3*f–g″*), microinjected cell clones were clearly distinguishable by expression of the histone H2B-RFP reporter in both NTC and *Ddx21*-specific siRNA microinjection groups. However, whereas DDX21 protein was expressed in all cell nuclei (specifically nucleoli) in the NTC control group, it was only readily detected in cell nuclei derived from the non-microinjected clone in the *Ddx21* targeted group and appeared highly, if not completely, reduced in the marked microinjected clone (figure 3*g,g′*,*g″*). Indeed, normalized quantitation of DDX21 immunofluorescence confirmed a highly significant reduction in overall average expression per cell (encompassing both microinjected and non-microinjected cell clones) in the *Ddx21* siRNA treatment group versus the NTC control (figure 3*c*–*e*; electronic supplementary material, figure S4a–c). This was true of both outer and inner cell populations. Generally, DDX21 expression was higher in outer versus inner cells in the NTC siRNA controls and was therefore consistent with observations made on similar late blastocysts treated with vehicle control DMSO (figure 2; electronic supplementary material, figure S2). The *Ddx21* siRNA-induced reduction in DDX21 protein expression was most robust in the marked microinjected clone in both inner and outer cells (i.e. when compared with either the non-microinjected sister clones of the same embryos or the equivalent microinjected clone of the control NTC siRNA treatment group; figure 3*e*). Thus, confirming the anticipated cell-autonomous effect of the microinjected *Ddx21* siRNA. However, it was observed that clonal *Ddx21* knockdown also affected DDX21 protein expression in the non-microinjected clone, as it was also significantly reduced (in both inner and outer cells) in *Ddx21* versus NTC siRNA treatment groups (figure 3*d*,*g″*). This demonstrates additional DDX21-related non-cell-autonomous effects that nevertheless did not affect every cell within that clone (figure 3*d*,*g′*). Notably, the microinjection of NTC siRNA had no statistically significant effect on DDX21 protein levels in either inner or outer cells and confirmed the absence of bias that could be attributable to the microinjection procedure. Hence, microinjection of *Ddx21* siRNA in one blastomere at the 2-cell stage causes efficient DDX21 protein knockdown within the clonal progeny of the microinjected cell but also results in reduced DDX21 expression in the accompanying non-microinjected sister clone, that is not restricted to the nucleolus by the late blastocyst stage.

The inspection of the embryos derived from the clonal NTC and *Ddx21* siRNA treatment groups also revealed profound morphological differences. Whereas the NTC group comprised embryos with typical late blastocyst morphology, the *Ddx21* siRNA microinjected embryos were typically smaller, with deformed blastocyst cavity and appeared to comprise much fewer cells (figure 3*f*,*g*). Although, in both NTC and *Ddx21* siRNA microinjected embryos, CDX2 protein expression was appropriately confined to outer cells only. Indeed, measuring the volume of the derived blastocysts, we observed a more than 50% reduction in *Ddx21* clonally downregulated embryos compared to control embryos (figure 3*j*). This result resonates with our recent report in which we revealed a similar blastocyst expansion defect, that is associated with significantly impaired PrE differentiation, upon p38-MAPKi (E3.5–E4.5) [11] and the fact we have identified DDX21 as a potential p38-MAPK effector (electronic supplementary material, table S1). An analysis of the average number, relative spatial positioning and clonal origin of cells showed no significant differences between the NTC siRNA microinjected clone and its non-microinjected counterpart. However, in the *Ddx21* siRNA group, only 27.45% of overall cell numbers originated from the microinjected blastomere compared to 49.6% for NTC microinjected embryos. Comparative cell number deficits in the microinjected (versus non-microinjected) clone were significant in both outer and inner cell populations, on average 8.31 versus 20.85 and 2.27 versus 7.08, respectively (figure 3*h*,*i*; electronic supplementary material, figure S4d). The reduced contribution was particularly stark for inner cells, averaging at 24.3% of the total ICM (compared to 46.2% in controls), that in absolute numbers only represents an average of 2.27 inner cells. In addition to such *Ddx21* siRNA microinjected clone-specific cell deficits, when compared with the equivalent clone in the control, we also observed significantly reduced numbers of outer and inner cells in the non-microinjected clone, on average 20.85 versus 26.67 and 7.08 versus 11.73, respectively (figure 3*h*,*i*). These data confirm both cell autonomous and non-autonomous effects of clonal *Ddx21* knockdown in developing mouse embryos and agree with the atypical non-nucleolar localization of DDX21 in the non-microinjected clone. Thus, clonal downregulation of *Ddx21* results in a global effect on embryonic development, effecting cell proliferation and blastocyst volume.

### 2.4. *Ddx21* downregulation results in defective blastocyst cell fate specification

**Figure 4.**
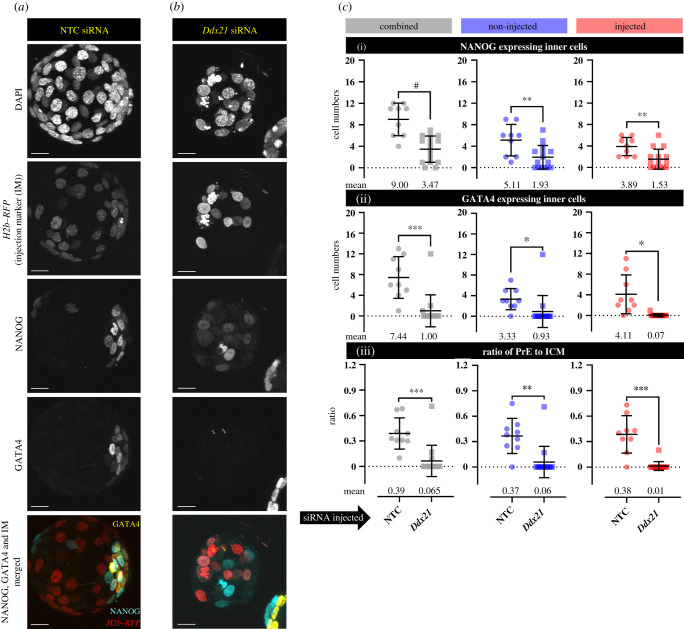
Effect of *Ddx21* knockdown on epiblast (EPI) and primitive endoderm (PrE) lineage specification at E4.5. (*a*,*b*) Confocal microscopy *z*-series projections of late blastocyst (E4.5) stage embryos derived from clonal microinjections (one blastomere at the 2-cell stage) of (*a*) control NTC siRNA (*n* = 9) and (*b*) *Ddx21* siRNA (*n* = 15) embryos immunostained for EPI (NANOG) and PrE (GATA4) lineage markers; note H2B-RFP distinguished microinjected and non-microinjected cell clones (experimental design in figure 3*a*). In merged images, NANOG, GATA4 and H2B-RFP signals are pseudo-coloured cyan, yellow and red, respectively. Scale bar, 20 µm. (*c*) Scatter plot quantification of (i) EPI cell numbers (inner cells only expressing NANOG), (ii) PrE cell numbers (inner cells only expressing GATA4), and (iii) the PrE to total ICM ratio, of clonal NTC siRNA and *Ddx21* siRNA microinjected injected embryos, as observed in either the microinjected or non-injected clones and both clones combined. Collated individual embryo cell number quantifications are provided in electronic supplementary material, data table S5. (Alternative comparative representations of these data are provided in electronic supplementary material, figure S5.)

Having previously confirmed a role for p38-MAPK during blastocyst maturation and PrE lineage differentiation [11–13] plus the effects of p38-MAPKi on DDX21 protein localization (figure 2) and the remarkably reduced contribution of *Ddx21* siRNA-injected blastomeres towards inner cells (figure 3), we wanted to assay the effect of clonal *Ddx21* knockdown on late blastocyst (E4.5) ICM cell fate. Accordingly, we repeated our clonal NTC/*Ddx21* siRNA 2-cell-stage microinjection experiments (as described in figure 3*a*) and assayed the expression of NANOG and GATA4 protein as markers of the EPI and PrE, respectively. Consistently, we again observed robust and significant decreases in overall, outer and inner cell numbers in *Ddx21* siRNA microinjected embryos, that was most evident in the microinjected clone but also present in the non-microinjected clone (electronic supplementary material, figure S5). We also observed significant and marked reductions in the overall number of ICM cells expressing either NANOG or GATA4, in both microinjected and non-microinjected clones, in *Ddx21* siRNA-treated embryos (figure 4*a–c*). While these reductions can reflect overall reduced cell number, it is notable the effect was stronger for GATA4 expressing PrE versus NANOG-positive EPI-like cells, with an overall average of 1.0 GATA4 expressing cell in *Ddx21* downregulated embryos compared to 7.44 in control NTC siRNA embryos (figure 4*c*(ii)). The equivalent NANOG expressing EPI cell numbers being 3.47 and 9.0, respectively, (figure 4*c*(i)). Moreover, among the *Ddx21* downregulated embryos, we observed an average of 0.93 GATA4 expressing cells originating from the non-injected blastomere and only 0.07 originating from the injected one (figure 4*c*(ii)). The equivalent NANOG expressing EPI cell numbers being 1.93 and 1.53, respectively (figure 4*c*(i)). Hence, despite the reduced number of overall, and specifically ICM, cells associated with clonal *Ddx21* downregulation, it is the derivation of GATA4 expressing PrE lineage that is markedly impaired by the late blastocyst stage. This is further revealed by the significantly reduced ratio of GATA4-positive PrE cells versus total ICM cell number, in both the microinjected and non-microinjected clones, of *Ddx21* siRNA-treated embryos (figure 4*c*(iii)); a trend not observed in the calculated ratio of NANOG-positive EPI-like cells in the same embryos (electronic supplementary material, figure S5(iv)). These data support a role for DDX21, potentially regulated by p38-MAPK activity, in facilitating PrE differentiation in the mouse blastocyst.

## 3. Discussion

The functional role of p38-MAPKs in preimplantation mouse embryos is a developing story and work from a few laboratories, including ours, have only begun to uncover the many facets of early development that this signalling pathway touches. In the earlier developmental stages, p38-MAPK activity is associated with the formation of filamentous actin [17] and embryo compaction and subsequent appropriate functioning of the TE to enable expansion of the blastocoel cavity [15,16]. Our own p38-MAPK research has primarily focused on the post E3.5 cavitated stages, thus specifically addressing blastocyst maturation and ICM specification towards EPI and PrE lineages [11–13]. In our recent work, we have identified a protein translation-associated regulatory role for p38-MAPK that underpins PrE cell fate differentiation. This role appears to be functionally upstream of the mTOR pathway and largely independent of the classically described FGF4-ERK-mediated mechanisms of PrE specification [11]. Whereas the upstream regulators/activators of this p38-MAPK pathway are the subject of informed speculation, potentially involving FGFR2, FGFR3 and/or PDGFRa [11], the downstream effectors can be many and encompass various mechanistic ontologies (reviewed in [39]). Hence, the identification of known factors involved in rRNA metabolism and ribosome biogenesis (for example, MYBBP1A [11] and DDX21 described here) as being sensitive to p38-MAPK signalling and essential in appropriate preimplantation embryonic development, further reinforces the emerging importance of the broader hypotheses of germane regulation of protein translation. Unlike mouse, human preimplantation embryonic development and blastocyst lineage specification is not dependent on the FGF-MEK pathway [40,41], and key signalling pathways involved are yet to be understood. Similarly, the role of rRNA processing and ribosome biogenesis-related factors in human peri-implantation development is rather limited too. Evidence from studies of HTATSF1 using both mouse preimplantation embryos and human embryonic stem (ES) cells (and embryoid bodies) demonstrates similar functional roles in both systems [8]. Since DDX21 and HTATSF1 are functionally related [8,20], it can be speculated that DDX21 is also functionally similar and relevant in human peri-implantation embryonic development.

Expression of the *Ddx21* gene is reported to start around the 2- to 4-cell stage and peaks at the 8-cell stage in mouse embryos, subsequently remaining high across all cellular lineages; with close to nil expression in the maturing oocyte (electronic supplementary material, figure S1) [34,35]. The protein appears to be expressed only after ZGA onset, and is thus not of maternal origin, becoming robustly evident in some 16-cell-stage nuclei. Around blastocyst formation, all cells express DDX21 protein that then exhibits a typical nucleolar localization as the blastocyst matures to the peri-implantation stage (figure 1*a,d*). Thus, the protein expression timeline closely follows that of the gene transcript. Moreover, the fact *Ddx21* knockdown clones only contribute an average of 11.69 cells to the late blastocyst, compared to 36.67 cells from the NTC siRNA microinjected blastomeres (figure 3*h*,*i*), strongly suggests DDX21 expression is ordinarily necessary for transition beyond the 32-cell stage and in turn blastocyst formation. This is reinforced by the observation that such cell-autonomous clonal cell number effects are also accompanied by non-cell-autonomous effects within the non-microinjected clone; implying the existence of inter-blastomere proliferative signals that are impinged upon by functional DDX21. The non-cell-autonomous phenotype of *Ddx21* downregulation remains to be mechanistically examined. Non-cell-autonomous mechanisms are functional in typical preimplantation development. For example, the expression of NANOG and SOX2 in mouse blastocyst EPI progenitor cells is required for PrE cell differentiation, via expression and secretion of FGF4 [42–46]. However, studies of nucleolar factors in preimplantation development, such as *Ddx21*, remain limited. Functional DDX21 is required for ribosome biogenesis and normal translation [20,22], and thus can possibly influence synthesis of paracrine factors during preimplantation development, consequently giving rise to the observed non-cell-autonomous effect. Furthermore, DDX21 also acts as a sensor of nucleotide stress and can regulate transcription [25], which can also have theoretically similar non-cell-autonomous effects. Future mechanistic studies are likely to shed more light onto the mechanism behind this phenotype.

The existence of such DDX21-related non-cell-autonomous effects is also suggested by the fact DDX21 protein expression in the non-microinjected clone of *Ddx21* siRNA microinjected embryos does not always exhibit nucleoli restricted expression typical of the blastocyst stages. Nucleoli are subnuclear structures closely associated with ribosome biogenesis that gradually transit through extensive compositional, functional and structural transformations between oogenesis and the embryonic morula stage (reviewed in [47,48]). Briefly, as developmentally competent oocytes slowly initiate the inactivation of RNA pol I and II, their nucleoli gradually become denser and form atypical structures termed nucleolus-like bodies (NLB); a process that is accompanied by reducing levels of component nucleolar proteins and correlates with paused ribosome biogenesis. After fertilization, further atypical nucleolar bodies, termed nucleolus-precursor bodies (NPB), emerge but are then replaced by somatic cell-type nucleoli after the morula to blastocyst transition. The functional significance of these atypical nucleolar bodies during early development is itself an interesting topic, with some reports suggesting their requirement during early ZGA and as gene regulatory structures in later stages [48]. Our observations of exclusive nucleolar-restricted DDX21 protein expression are concomitant with the emergence of somatic cell-type nucleoli during the earliest stages of blastocyst formation. Moreover, that such exclusive nucleolar expression is dependent on active p38-MAPK during blastocyst maturation, a time in which p38-MAPK-mediated regulation of protein translation is required for PrE differentiation [11]. Thus, it is possible DDX21, potentially acting downstream of p38-MAPK regulation, is involved in the remodelling of early embryonic cell nucleoli, in order to sustain the morula to blastocyst transition and subsequent ICM maturation and PrE formation.

Related to the subject of DDX21 protein localization, the observed differential association of DDX21 with condensed mitotic chromosomes as a function of developmental stage is intriguing (i.e. associated together in pre-blastocyst stages and excluded in the blastocyst; figure 1*c*,*e*). There is a report suggesting DDX21 forms complexes with members of PPP family of phosphatases [32] and indeed PP1 and other members of PPP family are key cell cycle regulators [33]. Thus, this association may explain the observed DDX21 localizations in relation to mitotic chromatin but the stage-specific differential nature of such localization also implies any potential DDX21–PPP interaction is likely to be dynamically regulated during preimplantation development. Indeed, it is during blastocyst formation that mouse embryo mitoses revert to centrosomal control, after previously being acentrosomally regulated since fertilization (a murine-specific characteristic [49]).

Post-translational modification by acetylation is reported to reduce DDX21 activity [28]. Here, we report reduced phosphorylation of DDX21 at serine-218 (S218) in mouse blastocysts upon p38-MAPKi (electronic supplementary material, table S1). According to the phosphosite.org database [50], this site is mouse-specific and not conserved in either humans or rats, thus, function of DDX21 in human peri-implantation embryonic development is either not regulated by p38-MAPKs or at least not at the equivalent amino acid residue. However, in mouse, the detected site does conform with the consensus [S/T]P dipeptide phosphorylation motif typical of the proline-directed mitogen-activated-kinase superfamily [51]. Direct experimental evidence of p38-MAPK phosphorylation-mediated functional and/or locational regulation of DDX21 is lacking. However, based on our current observations, this potential mechanism remains a possibility; as exemplified by the shift to exclusively nucleolar DDX21 localization upon blastocyst formation, that is itself sensitive to p38-MAPKi (figures 1 and 2). Such shifts in DDX21 localization are also observed in genetic models of congenital ribosomopathies, such as TCS, DBA and SDS, and are present in the differentiated derivatives of ES cells harbouring the specific genetic mutations but not the originating pluripotent parental ES cell lines themselves [24]. Thus, the p38-MAPKi phenotypes we observe during blastocyst maturation are, at least in the context of DDX21 localization, similar to those translation defects underpinning described ribosomopathies. Moreover, coupled with our recent report of reduced translation and rRNA processing in blastocysts cultured under p38-MAPKi conditions [11], such ribosomopathy-based insights [24] may be able to contribute to an expanded picture of the role of p38-MAPK signalling during blastocyst maturation and specifically ICM cell lineage and PrE derivation.

Our analysis of the contribution of *Ddx21* downregulated cell clones to late blastocyst (E4.5) ICM lineages is an extension of our previous observations obtained under p38-MAPKi culture conditions [11–13], and is similarly characterized by a reduction in the number of GATA4 expressing PrE cells (figure 4). However, it is the severity of this deficit that distinguishes such *Ddx21* knockdown phenotypes, as almost no GATA4 expressing PrE cells, were found to originate from *Ddx21* siRNA microinjected clone and moreover such GATA4 deficits were also non-cell autonomously observed in the non-microinjected sister clones (figure 4*c*(ii)). We similarly observed significant reductions in NANOG expressing EPI-like cells and outer TE cells in *Ddx21* siRNA microinjected embryos (figure 4*c*(i); electronic supplementary material, figure S5), although, compared to the almost complete block in PrE derivation, these lineages were less severely affected. By contrast, the p38-MAPKi (E3.5–E4.5)-derived phenotype is more specifically centred on PrE cell differentiation, with only limited and insignificant decreases in the quantity of other late blastocyst cell lineages [11–13]. Hence, these data suggest DDX21 probably plays a more fundamental role in regulating blastomere proliferation at the morula to blastocyst transition (figure 1) and developmentally precedes the previously studied period of p38-MAPKi during blastocyst maturation (E3.5–E4.5) [11–13]. E3.5 is the onset of cavitation and irreversible TE specification [52] and it is not possible to study ultimate ICM cell fate in embryos exposed to p38-MAPKi before E3.5, as the embryos arrest development with around 32 cells and fail to cavitate [12]. This confirms the morula–blastocyst transition as acutely sensitive to p38-MAPKi and developmentally coincides with the observed apparent proliferative block of *Ddx21* knockdown embryos (figures 3 and 4), and the observed transition of DDX21 protein expression to an exclusively nucleolar localization (figure 1). It is likely that the *Ddx21* downregulation-induced reductions in cell proliferation are accompanied by cell death. Analysing possible apoptotic cells based on DAPI staining, we did not see any significant difference between NTC and *Ddx21* siRNA microinjected embryos (electronic supplementary material, figure S6). Since the number of cells in the *Ddx21* siRNA microinjected embryos itself is lower than NTC siRNA microinjected embryos (figure 3*h*,*i*), there is proportionally more apoptotic cells in the *Ddx21* downregulated embryos. Thus, though *Ddx21* downregulation-induced cell death cannot be completely ruled out, cell proliferation defects appear to be the major phenotype. Interestingly, clonal *Ddx21* knockdown is still associated with cavity formation, albeit impaired in comparison to controls (figure 3*j*). Thus suggesting that at least part of the pre-E3.5 administered p38-MAPKi 32-cell arrested development phenotype could have a DDX21-related root. In relation to p38-MAPKi phenotypes during blastocyst maturation (E3.5–E4.5), it is likely any effect of p38-MAPK-mediated phospho-regulation of DDX21 (figure 2) will probably not be as drastic as efficient downregulation of the *Ddx21*-derived mRNA and protein caused by RNAi. This leaves open the possibility active p38-MAPK regulation of DDX21 function is a contributing factor to our previously observed p38-MAPK role in protein translation, mouse blastocyst ICM maturation and PrE differentiation [11]. Much like our observations with *Ddx21* (reported here) and *Mybbp1a* [11] clonal downregulation in preimplantation embryos, *Mybbp1a* [18] and *Gnl3* (Nucleostemin) [29] homozygous gene knockouts also result in defective development by the late blastocyst stage. These collective observations are bringing into focus the indispensable and potentially non-redundant role of various ribosome biogenesis-related genes during the preimplantation stages of mouse development and the formation of the blastocyst ICM lineages in particular. Moreover, they also highlight the importance of the underpinning regulatory signalling mechanisms, such as the p38-MAPK pathway, responsible for their proper functioning.

## 4. Methods

### 4.1. Mouse lines and embryo culture

All mouse-related experimental procedures (i.e. collecting preimplantation stage embryos for further study) complied with ‘ARRIVE’ guidelines and were carried out in accordance with EU directive 2010/63/EU (for animal experiments). Superovulation and strain mating were used to produce experimental embryos, with F1 hybrid (C57Bl6 × CBA/W) females injected subperitoneally with 7.5 IU pregnant mare serum gonadotrophin/PMSG (Folligon, MSD Animal Health, Boxmeer, The Netherlands) and 48 h later with 7.5 IU recombinant human chorionic gonadotrophin/hCG (Sigma-Aldrich, St Louis, Missouri, USA; cat. no. CG10), followed by overnight F1 male mating (successful mating confirmed by the presence of vaginal plugs). E1.5 (2-cell) stage embryos were isolated from oviducts in M2 media (pre-warmed at 37°C for at least 2–3 h) and thereafter cultured in KSOM (Sigma-Aldrich, EmbryoMax KSOM Mouse Embryo Media; cat. no. MR-020P-5F—pre-warmed and equilibrated in 5% CO_2_ and 37°C) with amino acid (AA) supplementation; Gibco MEM Non-Essential Amino Acids Solution (100X) (Thermo Fisher Scientific, Paisley, Scotland; cat. no. 11140035) and Gibco MEM Amino Acids Solution (50X) (Thermo Fisher Scientific; cat. no. 11130036) were used to a working concentration of 0.5X. Embryos were cultured in micro-drops prepared in 35 mm tissue culture dishes covered with light mineral oil (Irvine Scientific, Santa Ana, CA, USA; cat. no. 9305), in 5% CO_2_ incubators maintained at 37°C until the appropriate stage. Pharmacological manipulations were performed by addition of chemical agents dissolved in dimethyl sulfoxide (Sigma-Aldrich; cat. no. D4540) to KSOM + AA and equivalent volumes of DMSO were used as vehicle controls. Note, the p38-MAPK (SB220025–20 µM) inhibitor concentration used was derived by empirical titrations previously employed by ourselves [12] and literature-based precedents [15,53]. All KSOM-based culture media, with or without additional chemicals (AAs, pharmacological agents), were pre-warmed and equilibrated in 5% CO_2_ and 37°C for at least 3-4 h prior to embryo transfer.

### 4.2. Sample collection for mass spectrometric analysis of differential (phospho)proteome

Two-cell (E1.5) stage embryos were cultured in normal KSOM + AA conditions until E3.5 + 2 h and thereafter 100 embryos each were moved to control or p38-MAPKi conditions and cultured for another 7 h (E3.5 + 9 h). The embryos were then washed through pre-warmed (37°C) Hank's balanced salt solution (HBSS, Sigma-Aldrich; cat. no. H9269) and lysed by moving to a 1.5 ml centrifuge tube containing about 15 µl of SDT-lysis buffer (4% (w/v) SDS, 100 mM Tris–HCl pH 7.6, 0.1 M DTT). Cell lysis was performed by incubating the tubes in a 95°C thermoblock for 12 min, brief centrifugation at 750 r.p.m., cooling to room temperature and storage at −80°C.

### 4.3. Samples preparation for liquid chromatography–mass spectrometry analysis

Individual protein solutions were processed and analysed as described previously [11]. Briefly, protein lysates were processed by the filter-aided sample preparation (FASP) method. FASP eluates were used for phophopeptides enrichment using High-Select TiO2 Phosphopeptide Enrichment Kit (Thermo Fisher Scientific, Waltham, MA, USA). LC-MS/MS analyses of peptide mixtures (not enriched and enriched in phosphopeptides using TiO2 enrichment kit) were performed using a RSLCnano system connected to Orbitrap Fusion Lumos mass spectrometer (Thermo Fisher Scientific) as previously specified [11]. Analysis of the mass spectrometric RAW data files was performed using the MaxQuant software (v. 1.6.1.0) using default settings. MS/MS ion searches were executed against the modified cRAP database (based on http://www.thegpm.org/crap, 111 protein sequences) containing protein contaminants like keratin, trypsin, etc. and UniProtKB protein database for *Mus musculus* (ftp://ftp.uniprot.org/pub/databases/uniprot/current_release/knowledgebase/reference_proteomes/Eukaryota/UP000000589_10090.fasta.gz; v. from 20 June 2018, number of protein sequences: 22 297). Other conditions were the same as previously described [11]. Protein intensities reported in proteinGroups.txt file and evidence intensities reported in evidence.txt file (output of MaxQuant program) were further processed using the software container environment (https://github.com/OmicsWorkflows), v. 3.5.3c. Processing workflow is available upon request. Protein and phosphopeptide candidates were selected based on the following criteria; differential phosphopeptide candidates (exhibiting greater than or equal to 1.5-fold differential abundance; note the cut-off was applied to acknowledge the potential sensitivity threshold limitations involved using such scare starting material) were selected based on the criteria described previously [11]. The mass spectrometry proteomics data have been deposited to the ProteomeXchange Consortium via the PRIDE [54] partner repository with the dataset identifier PXD025754.

### 4.4. Embryo manipulation by microinjections

Single (for immunofluorescence confocal microscopy) or double (for qRT-PCR) blastomere microinjections of 2-cell (E1.5) stage embryos were performed using the FemtoJet 4i (Eppendorf, Hamburg, Germany; cat. no. 5252000013) micro-injector, mechanical micro-manipulators (Leica, Wetzlar, Germany; cat. no. ST0036714) and CellTram Vario (Eppendorf; cat. no. 5176000033) pneumatic handler, under a negative capacitance enabled current controlled by an Electro 705 Electrometer (WPI, Sarasota, FL, USA; cat. no. SYS-705) and on the stage of an Olympus IX71 inverted fluorescence microscope. Embryos were pneumatically handled and immobilized for microinjection using a borosilicate glass capillary holder (without filament—Harvard Apparatus, Holliston, MA, USA; cat. no. 30-0017). Micro-injectors were connected to needles prepared from filamented borosilicate glass capillaries (Harvard Apparatus; cat. no. 30-0038) using a Narishige PC-10 capillary glass needle puller (Narishige Scientific Instrument Lab., Tokyo, Japan). siRNAs were co-microinjected at 10 µM concentrations, with 50 ng µl^−1^ *H2b-RFP in vitro* transcribed (mMESSAGE mMACHINE T3 IVT kit, Thermo Fisher Scientific; cat. no. AM1348) and poly-A tailed (Poly(A) Tailing kit, ThermoFisher Scientific; cat. no. AM1350) mRNA, in pre-warmed drops of M2 media overlaid with mineral oil, on the surface of concaved microscope slides. The non-targeting control siRNA (NTC) used was from Qiagen GeneGlobe (Qiagen, Hilden, Germany; cat. no. SI03650318) and the *Ddx21* siRNA used was from Thermo Fisher Scientific Silencer Select (Thermo Fisher Scientific; cat. no. 4390771 and assay ID: s80158).

### 4.5. Immunofluorescence staining, confocal microscopy and image acquisition

To remove the *zona pellucida*, preimplantation embryos at the appropriate developmental stage were quickly washed and pipetted in pre-warmed drops of acidic Tyrode's Solution (Sigma-Aldrich; cat. no. T1788) until *zona pellucidae* were visually undetectable and then immediately washed through pre-warmed drops of M2 media. Embryos were fixed, in the dark, with 4% paraformaldehyde (Santa Cruz Biotechnology, Inc., Dallas, TX, USA; cat. no. sc-281692) for 20 min at room temperature. Permeabilization was performed by transferring embryos to a 0.5% solution of Triton X-100 (Sigma-Aldrich; cat. no. T8787), in phosphate-buffered saline (PBS), for 20 min at room temperature. All washes post-fixation, permeabilisation and antibody staining were performed in PBS with 0.05% TWEEN 20 (Sigma-Aldrich; cat. no. P9416) (PBST) by transferring embryos between two drops or wells (of 96-well micro-titre plates) of PBST, for 20 min at room temperature. Blocking and antibody staining was performed in 3% bovine serum albumin (BSA; Sigma-Aldrich; cat. no. A7906) in PBST. Blocking incubations of 30 min at 4°C were performed before both primary and secondary antibody incubation; primary antibody incubation (in blocking buffer) was performed overnight (approx. 16 h) at 4°C and secondary antibody incubation carried out in the dark at room temperature for 70 min. Stained embryos were mounted in DAPI containing mounting medium (VECTASHIELD; Vector Laboratories, Inc., Burlingame, CA, USA; cat. no. H-1200), placed under coverslips on glass-bottomed 35 mm culture plates and incubated at 4°C for 30 min in the dark, prior to confocal imaging. Details of the primary and secondary antibody combinations used can be found in electronic supplementary material, table S2. Complete embryo confocal z-series images were acquired using an FV10i Confocal Laser Scanning Microscope and FV10i-SW image acquisition software (Olympus, Tokyo, Japan). Images were analysed using FV10-ASW 4.2 Viewer (Olympus) and Imaris X64 Microscopy Image Analysis Software (v. 6.2.1; Bitplane AG—Oxford Instruments plc, Abingdon, UK). Cells were counted both manually and semi-automatically using Imaris X64 encoded functions.

### 4.6. Cell number quantification, statistics and graphical representation

Total embryo cell number counts (plus outer and inner cell populations) from confocal acquired z-series micrographs (based on DAPI nuclei staining) were further subcategorized as inner or outer based on the absence or presence of CDX2 expression, respectively (figure 3). EPI or PrE cells were quantified based on detectable and exclusive expression of NANOG and GATA4 protein expression, respectively (figure 4). Cells not located within blastocyst ICMs that also did not stain for either GATA4 and/or NANOG were designated as outer/TE cells. Initial recording and data accumulation was carried out using Microsoft Excel and further statistical analysis and graphical representations were performed with GraphPad Prism 8. Based on the normality and lognormality comparisons, appropriate statistical tests were used for the compared datasets (summarized in electronic supplementary material, table S3). Unless otherwise stated within individual graphs as a specific *p*-value (if statistically insignificant), the stated significance intervals were as follows: *p*-value < 0.0001 (#), 0.0001–0.001 (***), 0.001–0.01 (**) and 0.01–0.05 (*). All graphs represent dot plots of the total sample size, with associated means and standard deviation error bars provided.

### 4.7. Fluorescence intensity quantification and statistical analysis

Levels of DDX21 (figures 2*e* and 3*c–e*; electronic supplementary material, figures S2a and S3a–c) and NUCLEOSTEMIN (GNL3) protein (figure 2*f*; electronic supplementary material, figure S2b) were quantified, using Fiji (ImageJ) [55], as fluorescence intensity per cell nucleus and differentiated into inner and outer cells based on the absence or presence of CDX2 immunofluorescence, respectively. The measurements settings for all of the above were as follows: Analyze > Set Measurements; and the following options were chosen: Area, Mean grey value and Integrated density. Using the ‘Polygon selections’ tool, individual cell nuclei were demarcated and the aforementioned measurements recorded. The selected area was then moved so as to encompass an area excluding that of the embryo or cell nucleus, respectively, and background measurements were recorded. This process was continued for all the embryos analysed, under both control and p38-MAPKi conditions and the results were transferred to a spreadsheet for further calculations. The corrected total cell fluorescence (CTCF), in arbitrary units, for each embryo was measured as such: CTCF = Integrated density – (Area of selected cell × Mean fluorescence of background readings) [56,57]—electronic supplementary material, data tables S1 and S2. The calculated CTCF are plotted as scatters, with mean and standard deviations marked. The CTCF differences were statistically tested using appropriate statistical tests (electronic supplementary material, table S3) based on normality and lognormality comparisons and the results, unless otherwise stated within individual graphs as a specific *p*-value (if statistically insignificant), are stated as following significance intervals: *p*-value < 0.0001 (#), 0.0001–0.001 (***), 0.001–0.01 (**) and 0.01–0.05 (*).

### 4.8. Blastocyst size and volume calculations

Equatorial outer circumference and total volume measurements for the blastocyst as a whole were carried out by measuring the embryo outer circumference of the centrally located widest Z-stack using Fiji (ImageJ) [55]. The measurements were set as follows: Analyze > Set Measurements; and the ‘Perimeter’ option was selected; using the ‘Polygon selections’ tool, the outer circumference was traced and measured. The radius of the measured circumference was deduced and that value was used to calculate an approximate volume for all the embryos analysed under both control (NTC) and *Ddx21* siRNA-injected conditions. The calculated volume in picoliters (pl) (tabulated in electronic supplementary material, data table S3) is plotted as a scatter, with mean and standard deviations marked. The differences were statistically tested using the Mann–Whitney test and the results, unless otherwise stated within individual graphs as a specific *p*-value (if statistically insignificant), are stated at the following significance intervals: *p*-value < 0.0001 (#), 0.0001–0.001 (***), 0.001–0.01 (**) and 0.01–0.05 (*).

### 4.9. Quantitative real-time PCR

Twenty embryos per condition (control (NTC) and *Ddx21* siRNA-injected) from global microinjection experiments were collected at E4.5 and immediately processed for RNA extraction and isolation using the ARCTURUS PicoPure RNA Isolation Kit (Thermo Fisher Scientific; cat. no. KIT0204), following the manufacturer's protocol. The entire eluted volume of total RNA was immediately DNase treated with TURBO DNase (Thermo Fisher Scientific; cat. no. AM2238) according to the manufacturer's provided protocol. The whole sample was then subject to cDNA synthesis using SuperScript III Reverse Transcriptase (Thermo Fisher Scientific; cat. no. 18080044), as directed by the manufacturer and employing oligo d(T)16 (Thermo Fisher Scientific; cat. no. N8080128), dNTP Mix (Thermo Fisher Scientific; cat. no. R0192) and RNase Inhibitor (Thermo Fisher Scientific; cat. no. N8080119). The synthesized cDNA was diluted as required with nuclease-free water and 1 µl was used in 10 µl individual final SYBR-green based qRT-PCR reaction volumes (PCR Biosystems Ltd, London, UK; cat. no. PB20.11) with oligonucleotide primers at a final concentration of 300 nM each. A Bio-Rad CFX96 Touch Real-Time PCR Detection System apparatus, employing standard settings, was used for data accumulation and initial analysis was performed with the accompanying Bio-Rad CFX Manager software. Triplicate measurements per gene were assayed from one experiment that were each technically replicated. The sequence of oligonucleotide primers (5*′*–3*′*) used were: (i) *Tbp* - GAAGAACAATCCAGACTAGCAGCA (sense) CTTATAGGGAACTTCACATCACAG (antisense) and (ii) - *Ddx21*: TTCCTTCTGCAACGGAAATAA (sense) and GAGGCACAGAATCCAAGAGC (antisense). The average transcript levels of the *Ddx21* gene were derived after internal normalization against *Tbp* mRNA levels. Data were acquired and initially analysed with CFX Manager, then processed in Microsoft Excel (biological and technical replicate averaging) and GraphPad Prism 9 (graphical output).

## Data accessibility

The mass spectrometry proteomics data have been deposited to the ProteomeXchange Consortium via the PRIDE partner repository with the dataset identifier PXD025754.

The data are provided in electronic supplementary material [58].
